# Young adults’ experience with using a web based digital resource to promote healthy preconception diet

**DOI:** 10.1186/s40795-025-01151-w

**Published:** 2025-10-23

**Authors:** Øverby NC, Ripatti KR, Valen EN, Hillesund ER

**Affiliations:** Department of Nutrition and Public Health, Faculty of Health and Sport Sciences, University of Agder, Kristiansand, Norway

**Keywords:** Diet and health, Preconception, Health promotion, Digital intervention

## Abstract

**Background:**

The nutritional status of both women and men before conception impacts growth, development, and long-term health of future children. Evidence suggests that a substantial proportion of young adults lack adequate nutritional preparedness for future pregnancy. We have developed a digital resource, PREPARED, targeting young adults’ diet, nutrition literacy and dietary behavior that is presently being evaluated in a randomized controlled trial. The aim of the present study was to examine young adults´ thoughts and experiences following use of the e-learning resource.

**Methods:**

We investigated whether the resource is perceived as useful by young adults (aged 20–35 years) and as practical support for obtaining a healthy diet. Nine young adults consented to participate and were given access to the resource for two months and were then interviewed using a semi-structured interview technique. Using a phenomenological approach the material was analysed by thematic analysis.

**Results:**

Three main themes were identified: *easier to make healthy choices*, *new justification for healthy eating*, and *further development*. Access to recipes with simple and well-known ingredients and easy-to-read articles with a nuanced and positive focus were decisive factors for inspiration and motivation to change diet.

Participants felt the resource saved time and appreciated its positive, informative approach. The emphasis on preconception nutrition was described as both enlightening and unfamiliar.

Still, the resource has potential for improvements like navigation on the website, easier recipe portion estimation, and requests for more text to read.

**Conclusion:**

According to young adults in this study, the PREPARED resource communicates clear and motivating messages about diet, health and the importance of nutritional preconception health for future parenthood. The resource was perceived as nuanced and trustworthy, which was highly valued. Our results inform future iterations of digital health tools or nutrition education programs for young adults.

**Supplementary Information:**

The online version contains supplementary material available at 10.1186/s40795-025-01151-w.

## Background

There is an increasing focus on the relevance of parental preconception health for the lifelong physical and mental health of future children [1], specifically studies find that parental preconception diet may influence child chronic disease risk and cognitive development [1–4]. Preconception health comprises both male and female health and concerns risk factors such as obesity, malnutrition, undernutrition, diabetes mellitus, which may all affect the metabolic, neurological and cardiovascular health and resilience of their offspring [1, 5]. In 2018, a Lancet series on preconception health reviewed existing preconception research from biological, epidemiological and behavioral perspectives and specifically highlighted the importance of adequate preconception nutrition for the health of the next generation [1, 2, 6]. Healthy diet and dietary behavior promote nutritional and metabolic health and prepares individuals for future pregnancy and parenthood [1]. Still, the preconception diet and nutritional health is not routinely on the agenda in Norway, where the main preconception focus is on folate supplementation and abstaining from alcohol for women planning for pregnancy [7]. We have previously identified and reported that young people are generally unaware of the importance of diet in preconception years for their future children’s health [8, 9].

National and local surveys suggest there is room for dietary improvement in the intake of fruits, vegetables, whole grains, nuts/seeds, fish and milk, as well as reduced intake of processed meat and salt among Norwegian young adults [10]. Valen et al. recently showed that the diet of Norwegian students is a reason for concern, showing low diet quality and less-than-optimal micronutrient intake as compared with recommendations, and a more deficient diet among men than women [11]. These studies indicate that a large proportion of young adults may not be nutritionally prepared for pregnancy [6, 11]. Internationally, suboptimal dietary behaviors are also reported. Data show that for UK, more than nine of ten young women reported consuming fewer than five fruit and vegetable portions daily [1]. A total of 80% of Australian women do not meet daily serves of vegetables [12].

Despite increased international acknowledgement of this phase within research, there is a lack of interventions for this crucial period. Traditionally, interventions targeting diet *before* parenthood have been aimed at women planning pregnancy or couples with reduced fertility. The Healthy Life Trajectories Initiative (HeLTI) study is one exception [13, 14], however their focus is on women solely, without addressing or targeting male preconception health. A recent systematic review identified ten *digital* interventions targeting weight, diet or physical activity in preconception years [12]. Of the identified interventions only two studies targeted the general preconception population with general dietary support, which is comparable to our study [15, 16], however with different approaches. Maas et al. evaluated a social marketing event with flyers, a website, TV- and social media approaches, targeting women in preconception age and health care personnel during a one week event [17]. Van Djik et al.’s digital intervention *Smarter Pregnancy* focus on risk factors regarding nutrition with tailored information, and targeted couples who planned for pregnancy [18]. Even though a few digital public health programs exists for the preconception phase, a focus on both men and women’s preconception diet is missing. Both Maas et al.’s and Van Djik’s study showed positive results, however data on how the participant experienced the interventions is not published.

In 2020 we launched the PREPARED study, a randomized controlled diet intervention aiming to improve young people’s diet and dietary behavior and evaluate potential long-term effects on future children’s health and development [19]. The PREPARED intervention comprises access to a digital resource with motivational messages, videos and texts highlighting dividends of healthy eating, illustrated recipes and practical information related to meal planning, food preparation and healthy cooking. All content is in line with official Norwegian dietary recommendations and underpins the relevance of food choice for health and well-being. Self-determination theory was used to guide promotional messages, aiming to meet participants’ need for autonomy, competence and relatedness [19]. The development of content was also supported by short interviews with people in the target group from Valen et al. [8].

To get insights into how young people perceive the relevance and usefulness of the digital resource, we invited young adults (aged 20–35 years) not included in the randomized controlled study to use the resource for two months, followed by an in-depth interview.

## Methods

### Aim

The aim of the present study was to examine young adults´ thoughts and experiences following use of the e-learning resource.

### Study population and recruitment strategy

Inclusion criteria for this study were men and women aged 20–35 years without biological children. This was the same age range as those included in our RCT, which was mostly chosen for practical reasons [19]. There were no exclusion criteria. We recruited participants through social media (Instagram and Facebook) and snowballing. Potential participants were provided with written information about the study and consented to participation through a study website. The second author (KRR) had many followers on her social media accounts, and we decided to post information about the study through these accounts. Following the first sharing of the post, eight people consented to participate. As we aimed for more participants, the posts were further shared by friends and family. In total 17 agreed to participate and were provided access to the digital resource for two months, which was one third of the access time provided in the main study. They did, however, receive notifications at an accelerated rate, with twice-weekly updates compared to weekly updates in the main study. After the two months of access, only nine of the 17 registered for the study (53%) accepted the invitation to be interviewed. There were eight female and one male participant. Their ages ranged from 21–32 years. They had a varied educational background. For more details see Table 2 under results.

### Interviews

A semi-structured interview guide (Supplement 1) was developed and used for the interviews. It contained three main sections: Introduction, main part of the interview, closing the interview. The introduction included a summary of the project and aim, additional information about anonymity, and the plan for the interview. The main part comprised of general questions about the extent of use of the digital resource and then focus on the following topics: 1) experience of participating in the study, 2) evaluation of the digital resource as a source of information, 3) motivation for dietary change based on new knowledge, and 4) thoughts of the usefulness of such interventions. The four topics were addressed in a natural manner: Can you tell me what you thought of the content of the digital resource? Questions and follow-up questions were prepared. Examples of prepared questions were: What did you think about the content of the articles on the website? What was it that you liked/did not like/can you say something more about it?

All interviews were conducted by KRR as part of her Master’s project. She piloted the interview guide with volunteers in line with Braun and Clarke (2013) [20]. Some minor changes in wording were made following the pilot interviews.

A total of nine interviews were conducted, with one man and eight women. All interviews were conducted digitally (by Microsoft Teams) to include participants from different counties. Each interview lasted from 10–25 min, largely depending on the extent to which the participant had used the digital resource. The interviewer sought to create a friendly and relaxed atmosphere. The interviews were recorded with an approved recorder from the University of Agder. The interviewer wrote down her impressions and observations immediately after the interview and identified ways to improve the interview technique [20]. No repeat interviews were done, and the transcripts were not returned to the participants.

### Data analysis

All interviews were transcribed by the interviewer shortly after interview completion and transcribed non-verbatim to provide essence and meaning of what the informant said. However, short breaks and words like mmm, uh, etc. were transcribed and included as they may provide nuance to the meaning of wording and sentences.

We took a phenomenological and inductive approach in the analysis, to derive meaning and create themes from the data without preconceptions. Thematic analysis was used in line with six analytic principles by Braun & Clarke (Table 1), aiming to identify themes and patterns of meaning across data. The principles include familiarization with the data, generating codes, searching for themes, reviewing themes, and defining and naming the themes. Two persons, KRR and NCØ, read the interviews several times, and discussed the codes, and themes together. ERH read parts of the interviews and participated in the discussions. One of the pillars of trustworthiness in qualitative research is credibility [21], which was enhanced in the interview and analysis process by a) the interviewer was young and could relate to the target population and understand their needs, b) acknowledging and reflecting upon personal biases. The interviewer was a master student in public health with special focus on healthy eating, having a personal aim in improving population diet. NCØ and ERH are professors of nutrition and had developed the course. All three reflected upon their own biases to uphold a neutral discussion regarding the results.

**Table 1:** Phases of the study‘s thematic analysis

Familiarizing with the data	This process started during the interviews and with taking notes. The transcripts were read by two of the authors
Generating initial codes	We generated initial codes and identified text that was relevant for each code. The whole data set was read and reread by two authors and codes were given. “Timesaving” is example of a code for the excerpt “…when everything is gathered in the same place it is much easier to find varied recipes than to search on the internet…”
Searching for themes	After the codes were identified, we collapsed codes into meaningful themes. Three main themes were identified
Reviewing themes	We reviewed themes and codes and the relationship between them. Some text was removed from one theme and added to another as the themes were more clearly stated in this phase
Defining and naming themes	We provided meaningful names to the themes
Producing a report	The report is the results presented in this paper

In line with Braun & Clarke, saturation was not aimed for [22], and the themes were identified as described in Table 1. The qualitative analysis software NVivo 13 was used for the analysis and two of the authors read all transcripts, while three of the authors participated in the analysis. COREQ checklist is used for the reporting [23].

## Results

The results represent the thoughts and experiences of the nine participants following two months of access to the PREPARED resource. An overview of the participants’ age, gender, education, pattern of accessing and use of the resource is presented in Table 2. There is little variation in age. We found no meaningful differences in the responses from the male participant compared with the female participants.

**Table 2:** Overview of informants’ age, sex, pattern of accessing of the resource and use of the resource

Informant	Age	Sex	Education	Access of the resource	Use of the resource
1	27	Female	Nurse and education in Sport Science	Following every notification by email	Read and viewed most of the content
2	27	Female	Not reported	Following every notification by email	Read and viewed most of the content
3	25	Female	Bachelor in economy and administration	On her own initiative	Primarily recipes
4	32	Male	Craft certificate	On his own initiative	Primarily recipes
5	24	Female	Student, criminology	Following every notification by email and sometimes on her own initiative	Read and viewed most of the content
6	24	Female	Personal trainer	On her own initiative	Mostly the videos
7	21	Female	Student, community planning	Following every notification by email	Primarily recipes
8	23	Female	Student, teacher education	On her own initiative	Primarily recipes
9	23	Female	Student, sport science	Following every notification by email and otherwise on her own initiative	Read and viewed most of the content

**Fig. 1:**
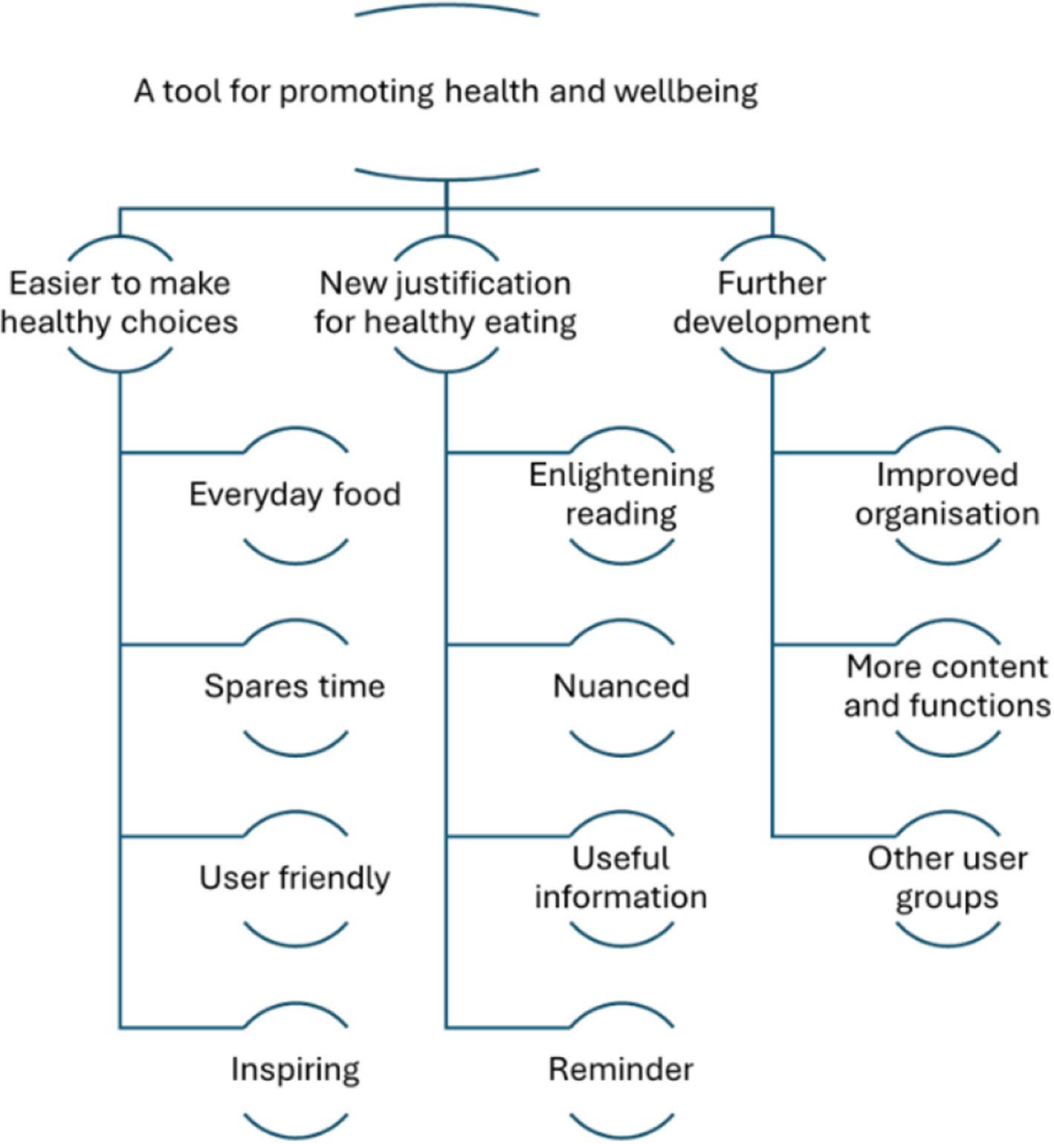
Overview of identified main and sub-themes

The overall impression from the interviews was that the PREPARED resource was perceived as a useful tool for promoting healthy eating. The analysis identified two main themes related to the thoughts and experiences young adults shared following use of the resource and one theme on suggestions for improvement of the resource (Fig. 1). The main themes are presented with their sub-themes in Fig. 1. Example quotes are provided to support the themes and summarized in Supplement 2. To distinguish the different informants when referring to quotes, they are identified in line with numbers in Table 2, Informant 1 (I.1) etc. and gender and age (I.1, female, age 27).

## Easier to make healthy choices

Based on informants’ reflections, the resource seems to be a tool that makes it easier to make healthy choices; “…*it has been a possibility for me to make things easier for myself since things have been planned for me. So, then it is actually just to make use of the tool*.” (I.9, female, age 23). Subthemes are presented below.

### Everyday food

Participants appreciated that the recipes were straight forward and perceived as easy-to-make. Many were of the impression that a healthy diet is associated with complicated recipes and ingredients that most people would not have in their kitchen. They highlighted the focus on “everyday food” as a very positive choice, like:

“*I think it was… with the recipes, that they are so straightforward and in a way something everyone can manage, which I think it is wise to focus on. Both the time required to make them and that the ingredients are well-known and that you don’t need too much kitchen utensils to make it*” (I.2, female, age 27), and “*I like that it is ordinary dinners and that you do not need a bunch of different things in your fridge. Many recipes entail a very long list of ingredients, but you use maybe half of it, while most of what is in the recipes here are… you buy fish burgers, hamburger bread and such*” (I.4, male, age 32).

### Spares time

Several pointed to time as a limiting factor in a busy everyday life and meant that time sparing solutions could be decisive in making the right choices: *“It is clear, and you get to know exactly what to buy. It is in a way a website that is time saving. You do not need to spend much time planning, and at least me and probably many more think it is boring to prepare food and boring to plan what to eat” (I.8, female, age 23).* Others expressed similar impressions: “…*it is motivating because you don’t have to move around and think and find out by yourself. So, you save some time”* (I.3, female, age 25) and *“It was easy and user friendly and not too time consuming, almost like you could go in and read almost the way you do in the newspaper”* (I.5, female, age 24).

### User friendly

The digital resource was easy to use and manageable: “*It is VERY easy. [..] You can reach all the recipes and then there is… ehm, for every recipe there is recipe and how to make it, not so much more… it is just simple and straightforward” (I.1, female, age 27).*

Regarding the recipe collection, participants valued the possibility of filters according to time use and complexity as well as suggestions for variations in the recipes. Vegetarian alternatives were valued specifically. “*It is very good that expected time use are listed and that something is easy, medium… that you can choose level of difficulty, for me who is lazy*” (I.1, female, age 27).

They liked the length and content of the information provided on the website: “*The papers were very interesting, and it was good that they were not long, but easy to read for us who do not know very much about these things. It was not too scientific if you understand. They were easy to understand*” (I. 5, female, age 24).

### Inspiring

The resource was described as inspiring and participants highlighted inviting pictures and the demonstration of easy ways of cooking and healthy eating:

“*I think it is easier to make healthy choices and include more vegetables. Ehm, I like to eat fruit on the go, but it is this thing with including vegetables in the dinner meal. That I think, has been easier with this website, and then you can also, ehm… if you know that you want a dinner with plenty of vegetables or want to eat healthier, it is easier to go in there to be inspired*” (I.8, female, age 23).

One commented on how the resource inspired him to make healthier choices:

“*I can guess what will be good for me to eat, but I need inspiration to actually do it, I may eat chicken and rice and all this stuff, but at the same time I want to make it more interesting than that, and this is what I have used this website for. I have started from something and maybe changed it somewhat to make it more exciting, but still in a healthy way” (I.4, male, age 32).*

The pictures of the actual dishes from the recipes were also perceived as motivational, like “*The pictures and stuff that were part of it were very inviting. You wanted to make the food, and that helped, at least for me who is not so fond of cooking or working in the kitchen” (I.8, female, age 23).*

## New justification for healthy eating

The website content had a different and new justification for healthy eating compared to what the participants normally encountered in information on diet and health. The new focus was perceived as enlightening and useful while at the same time being nuanced and working as a reminder.

### Enlightening reading

The content of the articles was described as enlightening in several ways, among others due to its focus, like “I feel that this was a little more updated knowledge”, (I.7, female, age 21) and continued as follows:

“…*there is a slightly different focus than those just focusing on losing weight, the articles are not only related to people who are overweight, but that it rather shifts focus… on… yes, other reasons for having a healthy lifestyle or… reasons for being conscious about eating healthily*” (I.7, female, age 21).

The same informant also talked about the underlying topic, that young adults’ preconception nutritional status may influence prospective children’s health. This was perceived as enlightening reading and not something known from before.

“*I think it was very interesting that this thing about… that what one eats also affects the next generation if you are going to have children, and that there are effects there. I have always heard that when one is pregnant, there are things one should not eat, but I have never heard anything about there being a process before that as well. This was actually very interesting, so I think that if more people gain more knowledge about it, that… as soon as you know that it affects others than just yourself, that is probably a motivation*” (I.7, female, age 21).

Another one put it this way: “*What was somehow new and maybe made me participate was related to this about having children and stuff, and how this is affected by diet*” (I.2, female, age 27). She added that it was the information on this topic she mostly noted.

### Useful information

The topics in the brief messages pointed to in the twice-weekly e-mails were in general perceived as useful by the participants.

“*You have good information gathered in one place and it seems like it comes from good sources compared to other things you read then, often. That, somehow, it is correct information and good. So, I think that many actually would have benefit of reading these messages*” (I.5, female, age 24).

“… *It has been useful for me even though it was information I probably knew of, but do not think of in everyday life. It is like, if one reads an article about wholegrain bread and how good it actually is for you, it leads to you choosing differently than one would have done if you hadn’t read that paper that day. The same pertains to fruit and vegetables”* (I.2, female, age 27).

### Nuanced

The information conveyed through texts were perceived as well-written and nuanced. One informant repeatedly mentioned that the knowledge conveyed seemed updated and relevant.

*“I feel that the information and messages are very updated and contrary to many other sources, especially digital newspapers, the focus is not only on dieting, overweight and what you should not eat and so forth. The focus is more on various groups in the population, and at the same time a focus on how it affects subsequent generations*” (I.7, female, age 21).

It was highlighted as positive that the digital resource did not focus on overweight and dieting, but rather on other reasons for healthy eating, and said: “*You see it everywhere. It is almost impossible to navigate the internet without finding papers saying, “skip this and you will lose so and so many kilos”, or “she did this to obtain the ideal body” *etc.*”* (I.4, male, age 32).

Informant 2 (female, age 27) described the information as nuanced: *“I think the articles were good and there was nothing that I sort of disagreed with… there is of course many opinions about diet. Sometimes you find things that you think that you do not agree with. I felt that this was a very nuanced focus”.*

### Reminder

Many of the informants had previous knowledge about diet and health. They nevertheless perceived the brief messages and articles as useful and as a reminder. Informant 2 (female, age 27) said: “*You get more conscious of this when you read about it once in a while, so it is a very good reminder in a way.”*

## Further development

The structure and amount of content could be improved according to participants. They asked for more functions, and improved functionality. They also suggested that the website might be useful for other groups than young people.

### Improved organisation

The website was perceived somewhat unstructured and participants mentioned that it could be difficult to find what they were most interested in reading. They requested easier ways to identify specific topics and navigate. “*If I clicked on a topic, more topics would open, but then it was difficult to go back, or… it was difficult sometimes to find the specific topic you wanted to read without going through other articles*” (I.9, female, age 23).

### More content and functions

More information was asked for, both in the weekly emails and what they encountered through the website. Like when responding to what could be improved one said, “*…ehm… or, really, maybe just that there was more. More articles that you could click on after watching the videos*” (I.6, female, age 24). One called for more information on how to use the website in a constructive way, saying: “…*if there was to be some kind of course or review that you had to go through* [..] *where you're told that in week 1 the focus is such and such. Then you get articles related to that theme and recipes. Really just the design of the website*” (I.2, female, age 27).

There were suggestions for more information on allergens in the recipes and better functions for the calculation of number of portions.

### Other user groups

Informants highlighted that the website would fit more target groups and could be relevant for people in all ages who are interested in improving their diet. They argued that it is easier for people to make healthier choices when the information is easily accessible.

“*I think for those who… whm… needs to be motivated to cook and be inspired and things like that, this is a very fine resource for them, and in a way. I think it is easier for people to choose healthier alternatives if they have access to recipes and stuff. And when everything is collected in one place it is much easier to find varied recipes instead of searching on the internet*” (I.3, female, age 25).

“… *as a student when you have this kind of website you can somehow plan and see – okay here are these ingredients. Since it is described what you need, you can plan what to make the next day so that you can use the same ingredients. Thinking of economy. That you get to use what you have in the refrigerator, as student you have to make plans to manage to use what you have in the fridge*” (I.8, female, age 23).

Informant 6, a personal coach (female, age24), thought that the previously commented everyday food was a factor that made access to the resource useful for more people.

“… *I think that for ordinary people it is very good to have this kind of website. There is so much… at least I can see it from my customers that, they actually believe 99% of what (they read) on the internet… but on this website is was a little more like… just eat ordinary food, not so very advanced stuff and yes… that it was very easy and plain, so I think such a website may make it easier for an ordinary/mainstream person… to take action and responsibility*” (I.6, female, age 24).

## Discussion

According to the young adults in this study, the PREPARED resource conveys trustworthy, easy-to-understand and inspiring messages about diet and health. The analysis indicates that use of the resource as a tool in everyday life made it easier and less time consuming to make healthy choices. The content of the motivational messages represented a new focus which was appreciated. There was room for improvement to make the website more user-friendly. Several suggested that the resource might be relevant and useful for a wider audience.

### Easier to make healthy choices

Participants’ original impression that preparing healthy meals would be time consuming with complicated recipes and lots of ingredients, was modified following exposure to the e-learning resource. The resource was acknowledged as a tool to be used in everyday life to make food planning and preparation easier. Participants especially valued the stock of recipes that covered easy-to-make, affordable dishes made from familiar ingredients. They also appreciated what they perceived as inspiring pictures and easy-to-understand descriptions. Although many were knowledgeable about what constitutes a healthy diet, they highlighted lack of time and inspiration as barriers to prioritise the planning and preparation of healthy meals. There are few digital interventions targeting preconception diet in general, and to our knowledge no qualitative analysis available. However, Scott et al. and Ku et al. have most comparable results to ours, as they have interviewed women in the preconception period, pregnancy and post pregnancy either after different lifestyle interventions [24] or before developing an intervention [25]. Both studies find that that perceived or real time scarcity may result in easier choices triumphing healthy choices, thus resulting in poor or less-than-optimal diet quality [24, 25].

Interestingly, having recipes and information compiled in one platform was highlighted by our participants as time-saving and inspiring, and seemingly helpful in bringing down barriers. One could speculate as to why participants originally thought cooking or eating healthily need to be time consuming. A recent paper by Jurado-Gonzalez et al. addressing Spanish students’ barriers for eating healthily, highlights that lack of motivation seemed to be the main barrier to healthy eating and home cooking, which was further influenced by the lack of cooking self-efficacy [26]. In addition to lack of knowledge and skills to prepare quick, affordable and nutritious meals, time scarcity was cited as a primary barrier and the authors propose context-specific culinary interventions that enhances students’ cooking self-efficacy as a measure to promote healthier eating habits [26]. The PREPARED intervention seems to have overcome some of these barriers by motivating young people to change their diet by inspiring text and providing easy recipes based on ordinary foods. Although not specifically mentioned by the respondents, it is tempting to speculate that use of the tool may have increased users’ cooking self-efficacy. Overall, our results may inspire future preconception diet interventions to include recipes that are uncomplicated, take short time to make and include inspiring pictures of the dish.

### New justification for healthy eating

When diet and dietary behaviour are discussed in the popular press it is often in relation to risk of disease or how what we eat may affect body weight [27]. The informants in this study seemed somewhat surprised by the broader and more nuanced focus of PREPARED.

They specifically mentioned the focus on preparedness for later parenthood as inspiring and new and a motivating factor related to dietary behaviour. Preconception health has not been a very prominent priority in public health policy but has gained more traction globally the last few years in line with developing knowledge [1, 2, 28]. In Norway, preconception care is still not an integrated part of primary health care, nor a priority in public health work. Our recent study showed that young adults are generally unaware of the concept of preconception health and the importance of diet in this phase [8]. Another study found that knowledge of the Developmental Origins of Health and Disease (DOHaD) is moderate in the young Norwegian population [9]. This is somewhat in contrast with Daly et al. who reported that within preconception knowledge, the role of *diet* is the factor that most young people are aware of [29]. The fact that preconception diet so far has not been a salient issue in Norway may explain the participants’ excitement over a new and different reason for eating healthily.

Another topic the participants found inspiring and new was the way diet is addressed in the e-learning resource. One can sense that currently the information that is being conveyed regarding diet today is related to reducing risk of obesity and various ways to lose weight. It is interesting that participants articulate an appreciation of the importance of diet for health and wellbeing per se, and in relation to the health and wellbeing for future children. To our knowledge few have addressed this issue, i.e., that nutritional importance is most often cited in relation to reducing risk of disease and obesity, and not as much for sustaining health, function and wellbeing. Kininmonth et al. have assessed how media present nutrition, confirming that it is most often cited in relation to obesity and CVD [27]. They also assessed the quality of newspaper articles on diet-related information and found that the lowest quality was found in papers on nutrition and obesity and that most had a sensational touch [27]. In our study, participants also state that the information provided is nuanced and seem to come from a credible source, which makes it trustworthy and useful. Future studies could benefit from these comments from young people, that they appreciate nuanced and non-tabloidized messages on diet.

It is worth commenting that the participants find that the regularly forwarded messages work as a reminder of what they know from before, and that reading it again motivates them for change. The current participants may have been specifically interested in diet, however, in Norway the school topic *food and health* is included in the school curriculum which secures a systematic food and health education [30]. Given that the school food and health education finishes in 9th grade (age 14–15 years), young adults may need a reminder or updated information about diet and food skills later in life along with starting a life on their own and being responsible for their own food choices.

It is interesting to note that most of the informants mention that the PREPARED resource might be interesting and relevant for other groups as well. This recommendation seems to stem from their experience of the resource being trustworthy, nuanced, providing a new focus and providing easy and motivating recipes that spares time. This seems to have motivated the participants to improve their diet, and to such a degree that they think it would be important for others as well.

### Further development

The participants had several suggestions for improvement, specifically regarding maneuvering the website. In hindsight we see that how to navigate the website should have been more clearly described in the notifications, and perhaps all information should have been present from the start and not built up in blocks of new information. Our thought was that correspondence with users or e-mail notifications may foster engagement with interventions [31, 32] and we therefore provided new information regularly. Several of the informants said that they only visited the webpage following e-mail notification about new content, indicating the importance of reminders. They also asked for more content, indicating a hunger for more knowledge and possibly more recipes. In a future version of this intervention, we will adjust in line with these users’ comments.

### Methodological limitations

This analysis is based on information from nine informants which was thought to be sufficient to respond to the overall research question [20, 33]. It may, however, be considered a weakness that participants were recruited narrowly, among others from one of the authors’ followers on Instagram. This Instagram account covers content related to exercise and diet and it is reasonable to think that its followers have a special interest in these topics. This may have resulted in a homogenous group and with limited representativity of the background population or the target group in the PREPARED study. They may, however, be representative of those who chose to participate in the PREPARED study, given that self-selection is a typical trait for this kind of intervention studies. Only one man was interviewed, meaning that the male voice is underrepresented. Thus, findings cannot be transferable to both genders. Recruiting less narrowly could have provided deeper insight into how the resource is perceived.

In the main study, the PREPARED RCT, participants had access to the website for 6 months, during which time they were exposed to 24 different topics. The interviewees in the present study were only exposed to the website for two months, however with double “speed”. They were therefore not exposed to all 24 topics as in the main study and may have lacked the global impression from the digital resource. It is sobering that they still seem to value the information they were exposed to and that they were able to spot the new focus that supports the aim of the overall project.

Even though the participants in the present study were generally positive about the resource and gave the impression that they used recipes quite extensively, we do not know whether dietary habits actually improved following its use. Effects of exposure to the resource will, however, be evaluated in the main study later. Further, as we only conducted interviews at one time point, we do not know how the participants experienced the continued use of the digital resource.

For trustworthiness of our results, credibility, transferability, dependability and confirmability should be discussed [21]. As commented under methods, measures were taken to increase credibility. More detailed, the interviewer was a public health master student with interest in diet and health. Her thoughts and preconceptions may have influenced how the interview guide was worded and the information interpreted. On the other hand, this may have facilitated important questions and opened for understanding of the information provided by the informants. Informants may have been familiar with the interviewer’s standpoints on various topics and wanted to agree with her. They may also have been influenced by her preconceptions. That said, it was clearly expressed in the interviews that no answers would be wrong, and that the interviewer only wanted their subjective opinion no matter positive or negative. The constructive inputs regarding topics they missed or wished had been a part of the website may indicate that they responded confidently and honestly. Regarding transferability, we have included descriptions of the population interviewed and how it was recruited allowing readers to judge whether this is relevant for their research. Since our data only include nine persons there is reasons to believe that other topics and themes might have been present had more people been included. Regarding dependability we have provided a step-by-step table of how the analysis was done in line with Ahmed [21]. Regarding confirmability, three persons have discussed the themes and codes, however, the interview objects themselves were not involved in the analysis, which would have improved the confirmability.

## Conclusions

In conclusion, overall, it seems that access to a collection of recipes with focus on simple ingredients which was considered time sparing, easy-to-read articles with a nuanced focus and inviting images were decisive factors for inspiration and thus motivation. This motivation seemed to promote a healthier diet and a wish to promote the resource to other groups of society. Based on findings from this study, we question whether dissemination of nutrition knowledge and fostering of food preparation skills towards the young is failing? The young ask for trustworthy, nuanced information, without always focusing on dieting or obesity risks. They also seem to lack food preparation self-efficacy and in general being provided with way too advanced food preparation information. Lastly, they acknowledge the value of knowing that what they eat might affect future children’s health. We owe it to the young population to find systems in which we provide nuanced info about diet and health. PREPARED may be one step on the way to providing such knowledge for young adults in general. The PREPARED intervention can be scaled up by being integrated into public health strategies at workplaces, university services or other settings where young people attend.

These lessons learned from this qualitative study will inform new interventions in the field of preconception diet and should also inform those conveying information about food and diet, like in primary care or public health nutrition messages. Still, there were only nine people interviewed in this study, and larger projects with a more diverse population would provide us with more insights on how this intervention works in a more varied preconception population.

## Supplementary Information

Supplementary Material 1.

Supplementary Material 2.

## Data Availability

The raw data used in the analyses presented in this paper are available from the corresponding author on reasonable request.

## Abbreviations

RCT: Randomized Controlled Trial
COREQ: Consolidated criteria for reporting qualitative studies

## References

[1] Stephenson J, Heslehurst N, Hall J, Schoenaker D, Hutchinson J, Cade JE, et al. Before the beginning: nutrition and lifestyle in the preconception period and its importance for future health. Lancet. 2018;391(10132):1830–41. 29673873 10.1016/S0140-6736(18)30311-8 PMC6075697

[2] Fleming TP, Watkins AJ, Velazquez MA, Mathers JC, Prentice AM, Stephenson J, et al. Origins of lifetime health around the time of conception: causes and consequences. Lancet. 2018;391(10132):1842–52. 29673874 10.1016/S0140-6736(18)30312-X PMC5975952

[3] Van Lippevelde W, Vik FN, Wills AK, Strömmer ST, Barker ME, Skreden M, et al. The impact of diet during adolescence on the neonatal health of offspring: evidence on the importance of preconception diet. The HUNT study. J Dev Orig Health Dis. 2021;12(5):798–810. 33256879 10.1017/S2040174420001087

[4] Jahan-Mihan A, Leftwich J, Berg K, Labyak C, Nodarse RR, Allen S, et al. The impact of parental preconception nutrition, body weight, and exercise habits on offspring health outcomes: a narrative review. Nutrients. 2024;16(24):4276. 10.3390/nu16244276 PMC11678361 39770898

[5] Dimofski P, Meyre D, Dreumont N, Leininger-Muller B. Consequences of paternal nutrition on offspring health and disease. Nutrients. 2021;13(8):2818. 10.3390/nu13082818 PMC8400857 34444978

[6] Barker M, Dombrowski SU, Colbourn T, Fall CHD, Kriznik NM, Lawrence WT, et al. Intervention strategies to improve nutrition and health behaviours before conception. Lancet. 2018;391(10132):1853–64. 29673875 10.1016/S0140-6736(18)30313-1 PMC6075694

[7] Helsenorge. Pregnancy. 2025. Accessed 6 Oct 2025. Available from https://www.helsenorge.no/en/pregnancy-and-maternity-carein-norway/.

[8] Valen EN, Øverby NC, Hardy-Johnson P, Vik FN, Salvesen L, Omholt ML, et al. Lessons learned from talking with adults about nutrition: a qualitative study in the PREPARED project. Matern Child Nutr. 2023;20:e13540. 10.1111/mcn.13540 PMC10765357 37277971

[9] Salvesen L, Valen EN, Wills AK, Hillesund ER, Vik FN, Engeset D, et al. Developmental origins of health and disease knowledge is associated with diet quality in preconception young adult men and women. J Dev Orig Health Dis. 2023;14(5):631–8. 38014542 10.1017/S2040174423000314

[10] Directorate of Health. Norkost 4 [online documment]. Oslo: Directorate of Health. 2024. accessed 6 Oct 2025. Available from: https://www.helsedirektoratet.no/rapporter/norkost-4. [In Norwegian]

[11] Valen EL, Engeset D, Øverby NC, Hillesund ER. Studentkost: a cross-sectional study assessing college students’ diets: reason for concern? J Nutr Sci. 2020;9:e39. 32983424 10.1017/jns.2020.33 PMC7503190

[12] O’Connor H, Willcox JC, de Jersey S, Wright C, Wilkinson SA. Digital preconception interventions targeting weight, diet and physical activity: a systematic review. Nutr Diet. 2024;81(3):244–60. 37845187 10.1111/1747-0080.12842

[13] Dennis CL, Marini F, Prioreschi A, Dol J, Birken C, Bell RC. The Canadian Healthy Life Trajectories Initiative (HeLTI) trial: a study protocol for monitoring fidelity of a preconception-lifestyle behaviour intervention. Trials. 2023;24(1):262. 37024918 10.1186/s13063-023-07271-7 PMC10079485

[14] Norris SA, Draper CE, Prioreschi A, Smuts CM, Ware LJ, Dennis C, et al. Building knowledge, optimising physical and mental health and setting up healthier life trajectories in South African women (Bukhali): a preconception randomised control trial part of the Healthy Life Trajectories Initiative (HeLTI). BMJ Open. 2022;12(4):e059914. 35450913 10.1136/bmjopen-2021-059914 PMC9024255

[15] Maas VYF, Poels M, Ista E, Menge LF, Vanden Auweele KLHE, de Bie RWA, et al. The effect of a locally tailored intervention on the uptake of preconception care in the Netherlands: a stepped-wedge cluster randomized trial (APROPOS-II study). BMC Public Health. 2022;22(1):1997. 36319990 10.1186/s12889-022-14343-x PMC9623982

[16] van Dijk MR, Koster MPH, Oostingh EC, Willemsen SP, Steegers EAP, Steegers-Theunissen RPM. A mobile app lifestyle intervention to improve healthy nutrition in women before and during early pregnancy: single-center randomized controlled trial. J Med Internet Res. 2020;22(5):e15773. 32412417 10.2196/15773 PMC7260659

[17] Maas VYF, Blanchette LMG, van Amstel W, Franx A, Poels M, Koster MPH. A social marketing strategy to promote preconception care: development of the Woke Women strategy. J Soc Market. 2022;12(2):154–73.

[18] van Dijk MR, Oostingh EC, Koster MPH, Willemsen SP, Laven JSE, Steegers-Theunissen RPM. The use of the mHealth program Smarter Pregnancy in preconception care: rationale, study design and data collection of a randomized controlled trial. BMC Pregnancy Childbirth. 2017;17(1):46. 28125970 10.1186/s12884-017-1228-5 PMC5270226

[19] Øverby NC, Medin AC, Valen EL, Salvesen L, Wills AK, Engeset D, et al. Effectiveness of a digital dietary intervention program targeting young adults before parenthood: protocol for the PREPARED randomised controlled trial. BMJ Open. 2021;11(12):e055116. 34853111 10.1136/bmjopen-2021-055116 PMC8638463

[20] Braun V, Clarke V. Successful qualitative research: a practical guide for beginners. London: SAGE Publications; 2013.

[21] Ahmed SK. The pillars of trustworthiness in qualitative research. J Med Surg Public Health. 2024;2:100051.

[22] Braun V, Clarke V. To saturate or not to saturate? Questioning data saturation as a useful concept for thematic analysis and sample-size rationales. Qual Res Sport Exerc Health. 2021;13(2):201–16.

[23] Tong A, Sainsbury P, Craig J. Consolidated criteria for reporting qualitative research (COREQ): a 32-item checklist for interviews and focus groups. Int J Qual Health Care. 2007;19(6):349–57. 17872937 10.1093/intqhc/mzm042

[24] Scott J, Oxlad M, Dodd J, Szabo C, Deussen A, Turnbull D. Creating healthy change in the preconception period for women with overweight or obesity: a qualitative study using the information-motivation-behavioural skills model. J Clin Med. 2020;9(10):3351. 10.3390/jcm9103351 PMC7603106 33086583

[25] Ku CW, Leow SH, Ong LS, Erwin C, Ong I, Ng XW, et al. Developing a lifestyle intervention program for overweight or obese preconception, pregnant and postpartum women using qualitative methods. Sci Rep. 2022;12(1):2511. 35169236 10.1038/s41598-022-06564-2 PMC8847557

[26] Jurado-Gonzalez P, Xavier Medina F, Bach-Faig A. Barriers to home food preparation and healthy eating among university students in Catalonia. Appetite. 2024;194:107159. 38103793 10.1016/j.appet.2023.107159

[27] Kininmonth AR, Jamil N, Almatrouk N, Evans CEL. Quality assessment of nutrition coverage in the media: a 6-week survey of five popular UK newspapers. BMJ Open. 2017;7(12):e014633. 29284712 10.1136/bmjopen-2016-014633 PMC5770895

[28] Stephenson J, Schoenaker DA, Hinton W, Poston L, Barker M, Alwan NA, et al. A wake-up call for preconception health: a clinical review. Br J Gen Pract. 2021;71(706):233–6. 33926884 10.3399/bjgp21X715733 PMC8087297

[29] Daly MP, White J, Sanders J, Kipping RR. Women’s knowledge, attitudes and views of preconception health and intervention delivery methods: a cross-sectional survey. BMC Pregnancy Childbirth. 2022;22(1):729. 36151510 10.1186/s12884-022-05058-3 PMC9508727

[30] Bjørkkjaer T, Palojoki P, Beinert C. Harnessing the untapped potential of food education in schools: nurturing the school subject Food and Health. Matern Child Nutr. 2024;20 Suppl 2(Suppl 2):e13521. 10.1111/mcn.13521 PMC10765364 37114411

[31] Brouwer W, Kroeze W, Crutzen R, de Nooijer J, de Vries NK, Brug J, et al. Which intervention characteristics are related to more exposure to internet-delivered healthy lifestyle promotion interventions? A systematic review. J Med Internet Res. 2011;13(1):e2. 21212045 10.2196/jmir.1639 PMC3221341

[32] Alkhaldi G, Hamilton FL, Lau R, Webster R, Michie S, Murray E. The effectiveness of prompts to promote engagement with digital interventions: a systematic review. J Med Internet Res. 2016;18(1):e6. 26747176 10.2196/jmir.4790 PMC4723726

[33] Guest G, Bunce A, Johnson L. How many interviews are enough?: an experiment with data saturation and variability. Field Methods. 2006;18(1):59–82.

