# Supplementary material for: Young adults’ experience with using a web based digital resource to promote healthy preconception diet
Source: BMC Nutr. 2025 Oct 23;11:194. doi: 10.1186/s40795-025-01151-w (PMC12548193; doi:10.1186/s40795-025-01151-w)
Supplement: Supplementary file 1 — Supplementary Material 1. [file 40795_2025_1151_MOESM1_ESM.docx]

# Supplement 1: Interview guide PREPARED

## Introduction

Thank you for participating and agreeing to be interviewed.

**Summary and aim**

This study is a part of my master’s in public health at the University of Agder, and as you might know the aim of this project is to explore young adults’ experiences with using a dietary e-learning resource. What we want to explore is what you thought about the recourse and whether it provided enough information and practical support for you to have a healthy diet. The information I get from those I interview will be used to develop the resource further, and the results will be published in an international scientific journal, so that other researchers can make use of the results and build on them.

**Anonymity**

The interviews will be recorded but will be deleted as soon as I have transcribed them. All data material will be anonymous, and nothing you say can be related to you. You can still withdraw at any time from the interview without any negative consequences for you.

**How the interview will be performed**

The interview will last for a maximum of 30 minutes. It is important to say that no answers are wrong, and we are not just wanting to hear positive feedback, negative feedback is equally important. And, as I have mentioned in my e-mail, it is ok if you have not used the website much, then there are reasons for that, that I would really like to explore.

## Main part of the interview:

**Background:**

-How often have you used the website?

-Via mail or own initiative?

-If the person has not used it much: What do you think can be the reason why you have not used it more?

**Main questions** are based on these main topics (not chronological): 1) Your experience of participating in the study, 2) evaluation of the website as an information site, 3) motivation for changing diet based on new knowledge and 4) thoughts about the usefulness of these kinds of interventions.

- What did you think about the concent of the articles on the website?
- What was it that you liked/did not like/can you say something more about it?
- Can you tell me what you thought about the recipe bank?
- What was it that you liked/did not like/can you say something more about it?
- Is there something in the resource that you have used more than other pages?
- Can you think of a reason for that?
- Have you been affected by something of what you have read or seen on the website, if so, in what way?
- Can you describe this more in detail? Do you have more examples?
- Has participating in this project affected your motivation for making more nutritious food/diet change?
- If so, in what way/can you tell me more about that?
- Can you describe a situation where you learned something new?
- Can you say a bit more about that/do you have more examples?
- Do you experience that diet and health knowledge is larger than what it was before?
- Can you say a bit more/do you have more examples?
- Can you say a bit about how it was to use the website, technically
- Can you say a bit about that/do you have more examples?
- Do you think that something could have been done differently with the resource (technically and content wise)
- Can you say a bit more about that/ Do you have more examples?
- What do you think about this website as an information source about diet?
- What do you think of the usefulness of such websites?

**The end of the interview**

- If you were to summarize your experience after using the resource, what would you say?
- Is there something that you feel that you have not said, or something that you think I should have asked you about?
- The last questions I would like to ask are about your age, education and if you have a job, what kind of job?

Again, thank you so much for participating.
