# Supplementary material for: Young adults’ experience with using a web based digital resource to promote healthy preconception diet
Source: BMC Nutr. 2025 Oct 23;11:194. doi: 10.1186/s40795-025-01151-w (PMC12548193; doi:10.1186/s40795-025-01151-w)
Supplement: Supplementary file 2 — Supplementary Material 2. [file 40795_2025_1151_MOESM2_ESM.pdf]

Supplement 2: Overview of all included quotes for the three themes and subthemes presented.

**Theme** *and subtheme*	**Quotes**
**Easier to make healthy choices**	*"...it has been a possibility for me to make things easier for myself since things have been planned for me. So, then it is actually just to make use of the tool."*
Everyday food	*"I think it was… with the recipes, that they are so straightforward and in a way something everyone can manage, which I think it is wise to focus on. Both the time required to make them and that the ingredients are well-known and that you don't need too much kitchen utensils to make it"*<br><br>*"I like that it is ordinary dinners and that you do not need a bunch of different things in your fridge. Many recipes entail a very long list of ingredients, but you use maybe half of it, while most of what is in the recipes here are… you buy fish burgers, hamburger bread and such"*
Spares time	*It is clear, and you get to know exactly what to buy. It is in a way a website that is time saving. You do not need to spend much time planning, and at least me and probably many more think it is boring to prepare food and boring to plan what to eat"*<br><br>*"…it is motivating because you don't have to move around and think and find out by yourself. So, you save some time"*<br><br>*"It was easy and user friendly and not too time consuming, almost like you could go in and read almost the way you do in the newspaper"*
User friendly	*"It is VERY easy. [..] You can reach all the recipes and then there is… ehm, for every recipe there is recipe and how to make it, not so much more… it is just simple and straightforward"*<br><br>*"It is very good that expected time use are listed and that something is easy, medium… that you can choose level of difficulty, for me who is lazy"*<br><br>*"The papers were very interesting, and it was good that they were not long, but easy to read for us who do not know very much about these things. It was not too*

	scientific if you understand. They were easy to understand"
Inspiring	*"I think it is easier to make healthy choices and include more vegetables. Ehm, I like to eat fruit on the go, but it is this thing with including vegetables in the dinner meal. That I think, has been easier with this website, and then you can also, ehm... if you know that you want a dinner with plenty of vegetables or want to eat healthier, it is easier to go in there to be inspired"*<br><br>*"I can guess what will be good for me to eat, but I need inspiration to actually do it, I may eat chicken and rice and all this stuff, but at the same time I want to make it more interesting than that, and this is what I have used this website for. I have started from something and maybe changed it somewhat to make it more exciting, but still in a healthy way"*<br><br>*"The pictures and stuff that were part of it were very inviting. You wanted to make the food, and that helped, at least for me who is not so fond of cooking or working in the kitchen"*
New justification for healthy eating	
Enlightening reading	"I feel that this was a little more updated knowledge"<br><br>*"...there is a slightly different focus than those just focusing on losing weight, the articles are not only related to people who are overweight, but that it rather shifts focus... on... yes, other reasons for having a healthy lifestyle or... reasons for being conscious about eating healthily"*<br><br>*"I think it was very interesting that this thing about... that what one eats also affects the next generation if you are going to have children, and that there are effects there. I have always heard that when one is pregnant, there are things one should not eat, but I have never heard anything about there being a process before that as well. This was actually very interesting, so I think that if more people gain more knowledge about it, that... as soon as you know that it affects others than just yourself, that is probably a motivation"*

	"What was somehow new and maybe made me participate was related to this about having children and stuff, and how this is affected by diet"
Useful information	*"You have good information gathered in one place and it seems like it comes from good sources compared to other things you read then, often. That, somehow, it is correct information and good. So, I think that many actually would have benefit of reading these messages"*<br><br>*"… It has been useful for me even though it was information I probably knew of, but do not think of in everyday life. It is like, if one reads an article about wholegrain bread and how good it actually is for you, it leads to you choosing differently than one would have done if you hadn't read that paper that day. The same pertains to fruit and vegetables"*
Nuanced	*"I feel that the information and messages are very updated and contrary to many other sources, especially digital newspapers, the focus is not only on dieting, overweight and what you should not eat and so forth. The focus is more on various groups in the population, and at the same time a focus on how it affects subsequent generations"*<br><br>*"You see it everywhere. It is almost impossible to navigate the internet without finding papers saying, "skip this and you will lose so and so many kilos", or "she did this to obtain the ideal body" etc"*<br><br>*"I think the articles were good and there was nothing that I sort of disagreed with… there is of course many opinions about diet. Sometimes you find things that you think that you do not agree with. I felt that this was a very nuanced focus".*
Reminder	*"You get more conscious of this when you read about it once in a while, so it is a very good reminder in a way."*
**Further development**	
Improved organisation	*"If I clicked on a topic, more topics would open, but then it was difficult to go back, or… it was difficult sometimes to find the specific topic you wanted to read without going through other articles"*

More content and functions	*"…ehm… or, really, maybe just that there was more. More articles that you could click on after watching the videos"*<br><br>*"…if there was to be some kind of course or review that you had to go through* [..] *where you're told that in week 1 the focus is such and such. Then you get articles related to that theme and recipes. Really just the design of the website"*
Other user groups	*"I think for those who… whm… needs to be motivated to cook and be inspired and things like that, this is a very fine resource for them, and in a way. I think it is easier for people to choose healthier alternatives if they have access to recipes and stuff. And when everything is collected in one place it is much easier to find varied recipes instead of searching on the internet"*<br><br>*"… as a student when you have this kind of website you can somehow plan and see – okay here are these ingredients. Since it is described what you need, you can plan what to make the next day so that you can use the same ingredients. Thinking of economy. That you get to use what you have in the refrigerator, as student you have to make plans to manage to use what you have in the fridge"*<br><br>*"… I think that for ordinary people it is very good to have this kind of website. There is so much… at least I can see it from my customers that, they actually believe 99% of what (they read) on the internet… but on this website is was a little more like… just eat ordinary food, not so very advanced stuff and yes… that it was very easy and plain, so I think such a website may make it easier for an ordinary/mainstream person… to take action and responsibility"*
